# Arbuscular Mycorrhizas: A Promising Component of Plant Production Systems Provided Favorable Conditions for Their Growth

**DOI:** 10.3389/fpls.2018.01329

**Published:** 2018-09-10

**Authors:** Michael Bitterlich, Youssef Rouphael, Jan Graefe, Philipp Franken

**Affiliations:** ^1^Department of Plant-Microbe Interactions, Leibniz-Institute of Vegetable and Ornamental Crops e.V., Großbeeren, Germany; ^2^Department of Agricultural Sciences, University of Naples Federico II, Naples, Italy; ^3^Department of Plant Physiology, Institute of Biology, Humboldt-Universität zu Berlin, Berlin, Germany

**Keywords:** arbuscular mycorrhiza, soil hydraulic properties, plant production, environment, atmospheric conditions

## Abstract

Arbuscular mycorrhizal (AM) fungi have become an attractive target as biostimulants in agriculture due to their known contributions to plant nutrient uptake and abiotic stress tolerance. However, inoculation with AM fungi can result in depressed, unchanged, or stimulated plant growth, which limits security of application in crop production systems. Crop production comprises high diversity and variability in atmospheric conditions, substrates, plant species, and more. In this review, we emphasize that we need integrative approaches for studying mycorrhizal symbioses in order to increase the predictability of growth outcomes and security of implementation of AM fungi into crop production. We briefly review known mechanisms of AM on nutrient uptake and drought tolerance of plants, on soil structure and soil hydraulic properties. We carve out that an important factor for both nutrient availability and drought tolerance is yet not well understood; the AM effects on soil hydraulic properties. We gave special emphasis to circular references between atmospheric conditions, soil hydraulic properties and plant nutrient and water uptake. We stress that interdisciplinary approaches are needed that account for a variability of atmospheric conditions and, how this would match to mycorrhizal functions and demands in a way that increased plant nutrient and water uptake can be effectively used for physiological processes and ultimately growth. Only with integrated analyses under a wide range of growing conditions, we will be able to make profound decisions whether or not to use AM in particular crop production systems or can adjust culture conditions in ways that AM plants thrive.

## Variable Plant Growth Responses to the Application of Mycorrhiza in Commercial Production Systems

In search for improvements in sustainability and resource use efficiency in agriculture, application of arbuscular mycorrhizal (AM) fungi has become an attractive tool given its known contribution to nutrient acquisition, pathogen resistance, and abiotic stress tolerance of plants (Rouphael et al., 2015). This ubiquitous symbiosis is characterized by low host specificity, but also by variable plant responses in experimental systems and plant production (Smith and Read, 2010). Inoculation with AMF under nursery conditions, in pots and in fields can increase plant growth and marketable yield of many horticultural crops (Regvar et al., 2003; Mena-Violante et al., 2006; Sorensen et al., 2008; Candido et al., 2013, 2015; Conversa et al., 2013; Colla et al., 2015), but this is not always the case (Bosco et al., 2007). Research studies revealed a mutualism-parasitism continuum in terms of symbiotic outcomes (Johnson et al., 1997). From an ecological point of view, the whole symbiotic continuum can underpin the resilience of symbiotic plants under particular environmental conditions. Reduced growth may allow for prolonged survival periods under resource-limited conditions, while enhanced growth may drive advantages for resource acquisition. In contrast to natural systems, growers seek to grow plants efficiently and maximize yields under given resources and economic inputs; hence, positive mycorrhizal growth responses are desired by growers. At the same time, growers are tasked with growing plants artificially and in protected environments. Likewise, plant scientists grow plants under engineered nutrient and water conditions, in restricted volumes, on constructed substrates, and at times under suboptimal climatic conditions. Those man-made conditions may be on the boundaries of evolutionary spectra or beyond, which can drive variability and unpredictability of growth outcomes under random application of AM fungi (Johnson et al., 1997) and hence, promote uncertainty rather than desired benefits for plant productivity. To confront that, one needs awareness that the application of AM fungi adds complexity to the cultivation system and advanced understanding is required of the growing conditions under which proper mycorrhizal function mitigates plant growth limitations.

Plant growth in particular environments is mostly determined by climatic conditions and the properties of the growing medium. As sessile organisms, plants have developed mechanisms for adjusting to variable environments in order to match resource supply and demand for survival and growth. AM fungi contribute to nutrient and water supply, but as obligate biotrophs, they also increase symbiotic carbon demands to develop hyphae and to complete their life cycle (Smith and Read, 2010). Hence, AM fungi application to production systems in particular, which are characterized by certain constraints to resource supply and demand, adds costs and benefits for plants. Since AM fungi change both resource supply and demand, it is likely that optimal growth conditions for symbiotic plants will differ from those required for non-symbiotic plants. All AM fungi have in common that they grow beyond the ambit of roots and can directly deliver nutrients to hosts. Besides direct nutrient delivery, they also change the properties of the growing medium (Rillig and Mummey, 2006; Rillig et al., 2010; Leifheit et al., 2014), which in turn alters constraints to nutrient and water extractability from this medium. The extent of AM effects in changing soil properties and their relevance to plant physiology and growth expectably differs with the underground habitat, i.e., physical and chemical properties and volume of soils and substrates (Querejeta, 2017), and the accompanied climatic conditions (Bitterlich et al., 2018b). This either allows effective usage of changes in supply or creates costs by superfluity of additional supply under low demand scenarios. Thus, integrative approaches that account for climatic conditions and edaphic properties are required to maximize and stabilize mycorrhizal growth outcomes.

## Nutritional Benefits and Drought Tolerance of Mycorrhizal Plants: Two Effects of the Same Origin?

The best understood characteristic of AM symbioses is the delivery of phosphorus (P) to plants from areas beyond root P depletion zones. Phosphorus is taken up by extraradical hyphae, transported toward the root and delivered to the plant via intracellular highly branched organs called arbuscules (Smith and Read, 2010). Since plant P uptake depends on diffusion and P has a low mobility in many soils, plant P uptake causes the formation of P depletion zones around roots, which are bypassed by fungal hyphae (Schachtman et al., 1998). Thus, AM plants become less dependent on the P solute concentration around roots. When soil moisture declines, water filled pores are emptied, reducing the diffusional cross sections and the extent of P depletion zones around roots (Gahoonia et al., 1994). Hyphae may cross P depletion zones and air-filled pore spaces, hence providing less tortuous pathways for P, making plant P uptake less dependent on soil moisture, and allow maintenance of higher P flow toward roots under drought.

For extraradical growth, AM fungi receive carbon from plant photosynthesis. Hence, with hyphal proliferation, carbon is redistributed to hyphae beyond root areas and into soils (Jakobsen and Rosendahl, 1990). The addition of organic matter and/or AM fungi into soils is known to contribute to soil structure, e.g., by entangling and enmeshing particles to aggregates, bridging of large voids, or by releasing organic matter during turnover (Miller and Jastrow, 2000; Rillig and Mummey, 2006). This in turn will modulate water retention (Rawls et al., 2003) and water and solute mobility, i.e., hydraulic conductivity (Durner, 1994), and thereby, water extractability for plants. Hitherto, the importance of AM effects on soil hydraulic properties for plant water relations is not well understood or characterized and will vary considerably with species identity, substrate properties, and rooting density. Substrate hydraulic properties influence the moisture stress that plants experience (Tardieu and Simonneau, 1998). The soil water status is sensed by roots via hydraulic and non-hydraulic signals (Tardieu and Davies, 1993); hence, apparent changes in soil hydraulic properties induced by AM fungi imply differential sensing of drought intensities by plants growing in AM and NM substrates given equal irrigation and/or during different time points in drying episodes. AM plants often grow better under moisture stress and AM-colonized substrates often have to dry more before host plants achieve a comparable physiological drought response (Augé, 2001). That may delay inhibition of transpiration and subsequently water flow in AM substrates (Bitterlich et al., 2018a,b).

In addition to putatively altered plant stress responses upon water retention and hydraulic conductivity modulations in AM substrates, AM colonized substrates may possess different water and solute flow resistances. If mycorrhizal effects on substrate hydraulic properties cause a reduction of flow resistance and/or an enhancement of water availability, two scenarios could be proposed: (i) AM plants either require fewer physiological adjustments to scavenge water and nutrients or, (ii) increased acquisition of water and nutrients facilitates resource deployment for growth while expending equal investments in physiological stress responses.

Both putative scenarios have the same prerequisites: AM fungi must colonize additional substrate volumes and, the plant must deliver the necessary resources for that. A feasible point of view would be that observed alterations of plant physiological responses in AM plants upon particular moisture levels are at least partially corollary effects of changes in substrate properties.

## Understanding Favorable Growth Conditions Can Underpin Stability of Growth Outcomes: an Integrated View

In nature and in plant production systems, continuous crop cycles face periodical transitions in growth limiting factors deriving from aerial and underground conditions. Conceptually, conditions under which the application of AM fungi leads to a mitigation of growth limiting factors are desired by growers. To elucidate those conditions, the fact that AM fungi are biotrophic soil microbes should be the basis. It is necessary to determine the underground constraints for nutrient and water availability, the variation of mycorrhizal impact on these constraints across different soils or substrates, and the corresponding atmospheric conditions that create a scenario where growth is limited by underground supply.

The increased independency of AM plants on P availability around roots can mitigate growth limitations under low P availability (Smith and Read, 2010). However, alleviation of P starvation in AM plants may not necessarily promote growth if analogous demands for nutrients other than P, especially nitrogen (N), cannot be satisfied. N and P have been shown to be the main nutrients regulating symbiotic intensity and growth responses and, insufficient N supply can lead to marked decreases in N/P ratios and relative N starvation in aerial tissues (Nouri et al., 2014). Although N may also be delivered by hyphae in non-negligible amounts (Govindarajulu et al., 2005), significant plant N acquisition (nitrate under most scenarios) is driven by plant transpiration, which induces mass flow toward the root and/or affects N diffusion indirectly (Oyewole et al., 2014). Vice versa, N availability determines mass flow driven acquisition of other nutrients (Matimati et al., 2013). This constitutes a circular reference to constraints set by substrate hydraulic characteristics and alterations in physiological moisture stress response (Matimati et al., 2013). Hence, direct (hyphal delivery) and indirect (substrate transport) AM effects in concert may be decisive factors in growth-limiting environments. A grower’s desire would then be growing conditions, where both ways of delivery are stimulated by AM fungi.

Frequently observed AM fungi-mediated changes to plant nutrient levels and drought stress responses (stomatal conductance) alter the capacity for leaf C assimilation (Augé, 2001; Augé et al., 2015). It is imperative that growth of AM plants should not be carbon limited, since photosynthesis also feeds the fungus. Indeed, it is known that low light intensities can be detrimental to positive growth responses in AM plants (Konvalinková and Jansa, 2016). This is logical, because apparent improvements in leaf nutrient status and CO_2_ availability (stomatal conductance) can become superfluous if the photosynthetic process is energy limited. Under such conditions, photosynthetic nutrient and water use efficiency would decline in AM plants. Then, AM fungi can constitute a carbon costly scenario at the expense of fungal demands (Konvalinková et al., 2015). Assuming photosynthesis is not energy restrained and leaf nutrient contents are high in AM plants, stomatal conductance should not limit photosynthesis. Vice versa, if nutrient contents are not affected and sufficient light is available, higher stomatal conductance in AM plants could alleviate CO_2_-imposed limitation to photosynthesis. Both scenarios can constitute a carbon gain scenario, which should be targeted by the grower. To close the circle, stomatal conductance will respond to substrate water potentials sensed by plant roots (Tardieu and Simonneau, 1998), which is determined partly by the water holding and transport capacity of the substrate. Such AM-induced alterations exhibit particularity for the combination of substrate, fungus, and plant.

## Mycorrhizal Effects on Substrate Hydraulic Properties May be Critical for Symbiotic Plant Growth and Nutrient-Water Uptake, but Their Actual Contribution Remains Unquantifiable

The effects of AM fungi on soil structure have been studied since decades and were reviewed extensively (Miller and Jastrow, 2000; Rillig and Mummey, 2006; Leifheit et al., 2014). These effects are specific to substrate and fungus–plant interaction and are mostly considered to originate from stabilization, formation (Leifheit et al., 2014), or even breakdown of aggregates, which induce changes to the secondary structure of soils. Besides AM effects on aggregation, AM fungi can have surfactant effects, i.e., they may change surface wettability and profiles of soil particles (Hallett, 2008; Rillig et al., 2010). AM fungi could also form micro-channels or contribute to the interconnectivity of pore spaces, by serving as a solid pathway for water across air spaces with direct contact to roots (Miller and Jastrow, 2000). The development of secondary structure and the other mentioned effects on soils contribute to soil water retention and hydraulic conductivity (Durner, 1992, 1994; Hallett, 2008), which are quantitative measures for underground water availability and transport. Water retention in rooted substrates has been shown to be affected by AM inoculation in different directions in soils and constructed substrates (Augé et al., 2001; Bearden, 2001; Augé, 2004; Daynes et al., 2013; Bitterlich et al., 2018a,b). Hitherto, only one study used root exclusion compartments to test whether hyphal ingrowth alone is sufficient to induce substrate water retention changes (Bitterlich et al., 2018a). Comparative studies that analyze AM effects in different substrates are missing, although physical reasoning implies that AM fungi-induced modifications of soil hydraulic properties will be substrate specific (Querejeta, 2017), depending on which soil or substrate matrix with its given properties is “offered” to AM fungi. Only recently, unsaturated hydraulic conductivity was observed to be enhanced in equally rooted substrates (Bitterlich et al., 2018b) and under root growth exclusion (Bitterlich et al., 2018a). To the best of our knowledge, those are the only studies that report AM influence on unsaturated hydraulic conductivity of substrates as a strict physical property independent of plant or fungal activity.

Water retention and hydraulic conductivity set constraints to plant water uptake and induce particular plant physiological responses to water shortage (Tardieu and Simonneau, 1998). In addition, water retention and hydraulic conductivity limit water and solute movement in the soil (Vogel et al., 2000). Feasibly, changes to soil hydraulic properties upon AM colonization could induce alterations in the physiological stress response of plants and, hence, induce subsequent changes to aerial plant gas exchange (Augé et al., 2001; Augé, 2004) and underground water and nutrient uptake.

Commonly observed is that AM plants require prolonged drying or more thoroughly dried substrates, before plants achieve a comparable physiological drought response (reviewed in Augé, 2001; Khalvati et al., 2005). In addition, non-hosts growing in a mycorrhizal soil showed improved stomatal conductance (Augé, 2004). And, whole plant transpiration of AM plants of equal size and root length densities was quantitatively limited by soil water flux, at higher drought intensity, i.e., substrate water potentials (Bitterlich et al., 2018b). The study showed that AM alleviated soil water flux limitations by enhancing hydraulic conductivity under higher drought intensities and by mitigating the decline in substrate water potential with water loss. Especially the latter cited studies imply that plants growing in a mycorrhizal substrate experience an underground environment that differs from non-colonized substrates in its hydraulic constraints (Augé et al., 2001; Augé, 2004; Bitterlich et al., 2018a,b). The result is that AM plants will respond with physiological adjustments to drought at varying soil water contents and/or consume resources in different time frames, which at least partially does not require direct alterations of their physiological state (Bitterlich et al., 2018b).

The vast majority of AM studies dealing with plant reactions to drought use distinct drought treatments, e.g., maintaining particular degrees of water contents/potentials during cultivation (e.g., Ruiz-Lozano et al., 1995; Porcel and Ruiz-Lozano, 2004), which are determined in advance (e.g., Subramanian et al., 1995; Porcel and Ruiz-Lozano, 2004), deliver a particular volume of water per irrigation cycle or withhold water for a certain time (e.g., Allen et al., 1981; Nelsen and Safir, 1982; Duan et al., 1996; Khalvati et al., 2005; Aroca et al., 2007; Ruth et al., 2011). However, indication mounts that soil water contents/potentials evoked by particular amounts of irrigation can diverge in AM and NM substrates after a certain time of cultivation (Bitterlich et al., 2018b). In particular, water contents do not need to correspond to equal water potentials or hydraulic conductivities in colonized and non-colonized substrates (Augé et al., 2001; Bitterlich et al., 2018a,b). This may change the physiological drought reaction in the plant. Moreover, a different physiological stress response in AM plants may induce shifts to mass flows in the substrate driven by altered transpiration (Bitterlich et al., 2018b), which in turn would affect mass flow driven nutrient transport (Matimati et al., 2013). Consequently, altered substrate drying rates induced by changes in substrate hydraulic conductivity or changed plant activity will result in different soil water contents. Subsequently, the resulting changes in soil moisture can lead to changes in water dependent nutrient diffusion coefficients, e.g., for P (Bhadoria et al., 1991; Gahoonia et al., 1994).

The effects of AM fungi on soil hydraulic properties will be substrate specific but are far from elucidated (Querejeta, 2017). At any rate, changes to hydraulic properties will partly determine the optimal choice for irrigation and fertilization practices that stimulate mycorrhizal benefits and avoid detrimental conditions for AM plants under given climatic factors. A mechanistic understanding of AM fungi-induced changes in hydraulic properties is therefore required and should be targeted in future research.

## Challenges Ahead

A positive AM-induced plant growth response will depend on the sum of events that constitute nutrient and water benefits by AM fungi, which can be efficiently used for carbon assimilation. Cultural practices for intensive plant production, normally absent in nature, impose risks to the safe use of AM fungi. Constraints to soil volumes, for example, hinder subsequent delivery of nutrients and water from the periphery and limit the explored volume. This may salvage economic risks caused by superfluity of application when AM effects become marginal, e.g., under high rooting densities and unfavorable growth conditions. Additionally, a more exhaustive behavior of AM plants can be induced when uptake is higher under particular growing conditions, which is less buffered by limited substrate volumes or not timely met by irrigation and fertilization (Bitterlich et al., 2018b). These shifts to time frames of resource exploitations in AM plants are dependent on atmospheric conditions and have to be known if growers want to adjust growing conditions to those in which AM plants thrive. Moreover, integrated approaches should be used that account for atmospheric conditions, plant and rhizosphere sizes and soil hydraulic properties. Mechanistic photosynthesis-based (e.g., Farquhar et al., 1980; Farquhar and Von Caemmerer, 1982) and quantitative plant-based uptake models (e.g., van Lier et al., 2013) are available, but have yet been scarcely applied to AM-studies (e.g., Boldt et al., 2011; Romero-Munar et al., 2017; Bitterlich et al., 2018b). Such models enable to quantify limitations of carbon assimilation and of water and nutrient uptake. Plant-based models also enable to analyze AM and NM plants of different sizes, because this is accounted for by implicit inputs. Knowing how substrate-borne limitations are mitigated by AM fungi and, under which conditions those mitigations become effective, will guide modulation of growing conditions to produce enlarged AM plants or enable decision making on whether or not to use AM fungi in a particular occasion.

## Author Contributions

MB wrote the manuscript. YR, JG, and PF critically revised the manuscript and contributed to writing.

## Conflict of Interest Statement

The authors declare that the research was conducted in the absence of any commercial or financial relationships that could be construed as a potential conflict of interest. The reviewer CP and handling Editor declared their shared affiliation.
